# Long-term prophylactic treatment and related real-world outcomes of patients with hereditary angioedema in Germany: A retrospective analysis of German claims and medical chart data

**DOI:** 10.1016/j.waojou.2026.101469

**Published:** 2026-09-22

**Authors:** Jens Greve, Nils Picker, Fabian Jülich, Verena Donatz, Ulf Maywald, Thomas Wilke

**Affiliations:** a Department of Otorhinolaryngology, Head and Neck Surgery, Ulm University Medical Center, Ulm, Germany; b GIPAM GmbH, Alter Holzhafen 19, Wismar, 23966, Germany; c CSL Behring GmbH, Germany; d payers GmbH, Germany; e IPAM e.V, Germany

**Keywords:** Angioedema, Hereditary, Drug utilization, Health care costs, Insurance claim review, Real-world evidence

## Abstract

**Background:**

Hereditary angioedema (HAE) is a rare genetic disorder characterized by recurrent swelling episodes that are potentially life threating and substantially impair quality of life. This study aimed to generate real-world evidence on the utilization of long-term prophylaxis (LTP), residual disease burden, and economic outcomes among patients with HAE in Germany.

**Methods:**

This retrospective cohort study examined LTP patterns and associated real-world outcomes using 2 anonymized German statutory health insurance datasets (AOK PLUS and GWQ) and a medical chart review (MCR) from 1 specialized HAE center. Claims datasets included patients initiating LTP between 2019 and 2023. The MCR provided complementary clinical information and was used to assess the plausibility of prescription-based estimates for attack frequency. Outcomes included LTP treatment sequences, HAE attacks requiring on-demand treatment (ODT), healthcare resource utilization and direct cost.

**Results:**

Across the claims datasets, 77 patients initiated first-line LTP: 30 (39.0%) with intravenous C1 esterase inhibitors (C1INH IV), 24 (31.2%) with lanadelumab, 16 (20.8%) with subcutaneous C1INH, and 7 (9.1%) with berotralstat. During follow-up, 27 patients (35.1%) switched therapy, most commonly from C1INH IV to lanadelumab. Despite prophylaxis, 64% of patients experienced at least 1 HAE attack requiring ODT, while 36% remained attack-free during the first year after LTP start. Median time to first treated attack ranged from 114 to 132 days. Prescription-based attack estimates were generally consistent with physician-documented attack frequencies but tended to yield slightly higher attack rates. Annual healthcare costs were driven primarily by outpatient medications, averaging €233,190 and €206,392 per person-year in the AOK PLUS and GWQ cohorts, respectively.

**Conclusion:**

This study provides real-world evidence on LTP use in Germany and demonstrates that a substantial proportion of patients continues to experience treated attacks despite prophylaxis. These findings highlight the importance of ongoing monitoring, individualized treatment optimization, and continued access to ODT.

## Introduction

### Disease and therapeutic background

Hereditary angioedema (HAE) is a rare genetic disorder characterized by recurrent, unpredictable episodes of swelling (angioedema) in various parts of the body, including the extremities, gastrointestinal tract, face, and airways. These attacks, which are often painful and debilitating, can be life-threatening when they involve the airways. The condition is mainly caused by a deficiency or dysfunction of C1 esterase inhibitor (C1INH), leading to uncontrolled activation of the complement system and dysregulated production of bradykinin, which induces angioedema.^1^ In patients with HAE and normal C1INH, similar clinical symptoms occur despite normal C1INH levels and function, and the condition is typically associated with pathogenic variants in genes such as F12, PLG, ANGPT1, KNG1, MYOF, HS3ST6, CPN1, or DAB2IP.^2^

Long-term prophylaxis (LTP) plays a critical role in the management of HAE by reducing the frequency and severity of attacks.^3^ Several therapies are available for LTP, including targeted therapies such as plasma-derived C1INH, kallikrein inhibitors (berotralstat, lanadelumab),^4^, ^5^, ^6^, ^7^ and the novel activated Factor XIIa (FXIIa) inhibitor garadacimab. These LTP options differ with regard to mechanism of action, route of administration, dosing frequency, and monitoring requirements, which may influence treatment selection, persistence, and switching decisions in routine clinical practice. Understanding treatment sequencing is therefore important for evaluating real-world management strategies and identifying unmet needs among patients with HAE. Furthermore, clinical trials have demonstrated that these LTP options are associated with reductions in HAE attack frequency.^3^^,^^8^, ^9^, ^10^ Additionally, in 2025, garadacimab was approved by the European Medicines Agency (EMA) as the first-in-class FXIIa inhibitor, based on results from the pivotal Phase 3 VANGUARD study, which demonstrated a significant reduction in HAE attack rates compared to placebo.^11^ However, given the retrospective design of this study, garadacimab was not yet included in the present analysis. In addition, as most available evidence originates from separate placebo-controlled trials, drawing robust conclusions regarding the comparative effectiveness of specific LTPs for HAE is constrained by the absence of head-to-head data. To address this, a recent network meta-analysis (NMA) indirectly compared the efficacy of available LTP options and found that efficacy varies across available LTP options.^12^ Garadacimab was associated with statistically significant benefits, including lower time-normalized HAE attack rates compared with berotralstat and lanadelumab administered every 4 weeks, fewer moderate/severe attacks compared with lanadelumab administered every 2 weeks, and greater improvement in Angioedema Quality of Life (AE-QoL) total score compared with berotralstat.

Although clinical trials have demonstrated the clinical efficacy and safety of currently available LTP therapies, evidence on their use in routine clinical practice remains limited. In particular, real-world data describing treatment sequencing, treatment persistence, switching patterns, healthcare resource utilization (HCRU), and the economic burden associated with LTP are scarce. Despite the availability of LTP therapies, breakthrough attacks may still occur, suggesting residual disease activity. It is unknown how many patients are affected by this in the real world as existing observational studies have largely focused on specific therapies or selected clinical outcomes only, and provided limited insight into how LTP therapies are utilized across the patient journey in routine care.^13^, ^14^, ^15^, ^16^ Furthermore, evidence from Germany remains sparse despite potential differences in treatment availability, reimbursement pathways, and healthcare delivery compared with other countries.^17^^,^^18^

### Rationale and study objectives

This study aimed to generate real-world evidence on the use of LTP among patients with HAE in Germany, Specifically, the study sought to describe patient and treatment characteristics, patterns of LTP use, including therapy sequencing and switching, HAE attacks requiring on-demand treatment (ODT), HCRU, and the economic burden associated with LTP in HAE.

To our knowledge, this is the first study to combine German statutory health insurance (SHI) claims data with physician chart review data to provide complementary real-world evidence on HAE. Claims data allow a comprehensive assessment of treatment patterns, HCRU, and costs associated with LTP use in patients with HAE as all healthcare sectors are covered in a longitudinal manner. The study further contributes to the limited evidence on assessing HAE attacks through German ODT prescription claims and comparing these estimates with clinically documented treated attacks from physician records.

## Materials and methods

### Data sources

This retrospective, descriptive cohort study utilized 3 data sources: 2 administrative claims databases and a single-site medical chart review (MCR) linking clinical chart data with diagnosis and prescription data as they are documented in claims datasets and, at the same time, available in the site's software systems. This research draws upon anonymized claims databases provided by GWQ ServicePlus AG and AOK PLUS (“Allgemeine Ortskrankenkasse”). The GWQ database consolidates claims data from 20 small- and medium-sized SHI providers and comprises up to 7.8 million observable insured individuals. In addition, the AOK PLUS claims dataset includes approximately 3.5 million insured individuals. Together, the datasets cover the period from January 2010 through December 2023. These datasets provided comprehensive information on patient demographics, comorbidities, medication history, HCRU, including hospitalizations and pharmacy dispensations. Additionally, AOK PLUS data uniquely included work absence records. Together, these datasets represent approximately 15% of the German SHI population, ensuring a broad and representative view of real-world healthcare utilization. While claims data offer high consistency, reliability and coverage of the whole inpatient/outpatient healthcare sector, they lack clinical information, such as attack frequency and severity. To complement the claims-based analyses, an MCR was conducted using medical records from a single specialist HAE practice in Germany. The MCR focused exclusively on patients who initiated LTP between 2020 and 2023, with data collected retrospectively via case report forms (CRFs). The claims and MCR datasets were analyzed independently and served complementary purposes. Claims data were used to assess treatment patterns, HCRU, and related costs, whereas the MCR provided additional clinical information not available in claims data, including HAE subtype, disease duration, physician-documented attack frequency, and attack characteristics. In addition, MCR data were used to assess the reliability of a prescription-based attack frequency measure. No patient-level linkage between the claims and MCR datasets was performed.

### Sample selection

#### Claims data

HAE cases were identified by using the German Modification of the International Classification of Diseases (ICD-10-GM) code D84.1 for defects in the complement system or T78.3 for angioneurotic oedema, which have been used in previous studies.^19^, ^20^, ^21^, ^22^ Individuals were defined as patients with HAE, if at least 1 primary inpatient diagnosis or 2 outpatient diagnoses in 2 different quarters within twelve months were observed between January 1, 2019 and December 31, 2023. Cases fulfilling the diagnosis selection criteria were required to be continuously insured for at least twelve months prior to the first observed diagnosis for HAE. Selected individuals were excluded from the analysis sample if they were younger than 2 years at the time of diagnosis with HAE or did not receive at least 1 HAE specific treatment as recommended by clinical guidelines, ie, plasma-derived C1 esterase inhibitors (C1INH; Anatomical Therapeutic Chemical [ATC] code B06AC01 or “Operationen-und Prozedurenschlüssel” [OPS] code 8–810. h), icatibant (ATC B06AC02 or OPS 6–005.4), conestat alfa (ATC B06AC04), lanadelumab (ATC B06AC05 or OPS 6-00c.8), or berotralstat (ATC B06AC06) in the entire study period. Patients with LTP treatment were defined as those with at least 1 prescription of drugs indicated for LTP in HAE (C1INH SC, berotralstat, or lanadelumab) or those receiving long-term use of C1INH IV (Cinryze or Berinert IV), which was defined as persistent treatment with C1INH IV for ≥183 days. As C1INH IV may be used both for LTP and ODT of acute HAE attacks, a duration threshold was required to distinguish sustained prophylactic use from intermittent or episodic administration. The ≥183 days-threshold was selected to identify treatment patterns consistent with routine prophylaxis, minimize misclassification of short-term prophylactic use and to align with established conventions used in previous claims-based analyses.^13^ LTP starters were followed from the first prescription date for an LTP treatment to date of death, loss to follow-up due to end of insurance, or end of the study period (December 31, 2023).

#### MCR

The MCR included patients aged ≥2 years with a confirmed HAE diagnosis at a single, specialized treatment center in Germany. Only patients who initiated LTP treatment between January 1, 2020, and December 31, 2023, were included. Medical records were retrospectively abstracted in January 2025, ensuring that all included patients had at least 12 months of follow-up available for analysis. A target sample size of 20 patients was set to provide clinically relevant validation of the claims-based approach while maintaining feasibility within the constraints of rare disease research.

### Study variables and statistical analysis

#### Patient baseline characteristics

Baseline characteristics were descriptively analyzed using summary statistics for continuous variables and frequency statistics for categorical variables, based on the index date (start of LTP treatment) or a 12-months baseline period ending on and including the index date. These included sociodemographic characteristics (age, sex, insurance status, HAE type), prevalent comorbidities, and medication history for treatment of HAE and related symptoms. For the claims data cohorts (AOK PLUS, GWQ), comorbidities were identified using ICD-10-GM codes (any diagnosis in inpatient and outpatient setting) and comprised frequencies of patients who were at least once diagnosed with hypertension, obesity, hyperlipidemia, and depressive episodes in the baseline period (for ICD-10-GM codes, see Table 1). Additionally, medication history related to acute HAE attacks was evaluated based on claims for drug prescriptions, with frequencies reported for patients who had received at least 1 prescription within 12 months prior to the index date. In the MCR cohort, detailed clinical information was extracted from available patient charts, including HAE subtype, disease duration However, certain clinical parameters, such as laboratory measurements, family history, and adverse events, were not consistently documented and therefore could not be systematically assessed across all patients.

**Table 1:** Baseline characteristics of patients on LTP treatment for HAE.

Parameter/Study cohort	AOK PLUS	GWQ	MCR
N Patients	41	36	20
**Sociodemographic characteristics at index date**			
Female sex – n (%)	30 (73.2)	25 (69.4)	11 (55.0)
Age at index date (in years) – mean (SD)	43.2 (18.5)	44.6 (17.8)	40.0 (15.1)
**Present comorbidities in baseline period (ICD-10-GM code)**			
Hypertension (I10–I15) – n (%)	10 (24.4)	9 (25.0)	n.r.
Hyperlipidemia (E78.0-E78.5; E78.80) – n (%)	5 (12.2)	9 (25.0)	n.r.
Obesity (E66) – n (%)	6 (14.6)	6 (16.7)	n.r.
Depressive episode (F32) – n (%)	7 (17.1)	8 (22.2)	n.r.
**Medication history related to HAE - n (%)**			
Plasma-derived C1INH	34 (82.9)	22 (77.8)	n.r.
Icatibant	20 (48.8)	16 (44.4)	n.r.

**Abbreviations:** C1INH, C1-Esterase inhibitors; HAE, hereditary angioedema; ICD-10-GM, International Classification of Diseases - German Modification; SD, standard deviation; n.r. Not reported

#### Treatment patterns

Descriptive statistics were applied to characterize the distribution of patients initiating LTP therapies. Start of LTP treatment was assumed based on the first observed prescription or administration of an LTP-specific medication, including C1INH IV, C1INH SC, lanadelumab, and berotralstat. For C1INH IV, persistent use for ≥183 days was additionally required to distinguish prophylactic use from ODT. Although Berinert IV is not approved in Europe for routine LTP, it was included to capture established real-world (off-label) use of C1INH IV prophylaxis in Germany.^7^^,^^23^ Time from initiation of the first LTP regimen to switch from the initiated regimen to another was evaluated, defined by a change in the prescribed LTP medication class. Median time to switch was estimated using Kaplan–Meier survival analysis, with censoring applied for patients without a switch event by the end of the study period or at death. In addition, stratification by line of treatment (first, second, and third LTP regimen since the start of observation) was conducted for the claims data cohorts (AOK PLUS, GWQ). Treatment switching patterns among patients receiving LTP were visualized using a Sankey diagram. Among patients receiving lanadelumab, the study examined the distribution of dosing regimens, categorizing patients into bi-weekly (336–365 defined daily doses per year [DDD/year]), every 4 weeks (168–196 DDD/year), alternating between bi-weekly and four-weekly use (197–335 DDD/year), and less frequent use (<168 DDD/year).

#### HAE attack frequency

In German claims data, HAE attack frequency is not directly recorded and therefore cannot be identified using diagnosis codes alone. To estimate the number of HAE attacks requiring treatment during active LTP therapy, dispensations for ODT (C1INH IV or icatibant) were used as a proxy. In this context, a proxy refers to an indirect measure that approximates a clinical event when the event itself is not directly observable in the data source. This approach was based on the assumption that ODT is generally prescribed and dispensed in response to acute HAE attacks requiring treatment. Because claims data do not capture therapeutic intent and C1INH IV may be used for both LTP and ODT for acute HAE attacks, C1INH IV prescriptions were excluded from the attack proxy during periods of long-term C1INH IV treatment (defined as ≥183 days of continuous use) to minimize misclassification of prophylactic treatment as ODT for acute attacks. Outside these periods, episodic C1INH IV use was retained as a marker of HAE attacks. Furthermore, multiple ODT dispensations occurring within a seven-day period were considered part of the same episode and counted as a single attack. This approach accounts for the possibility that a single HAE attack may require multiple administrations of ODT over several days, particularly in cases of prolonged or severe symptoms. Additionally, prescriptions for ODT issued more than 730 days apart were excluded, as these were assumed to represent replacement prescriptions following product expiration rather than treatment of an acute attack, given the shelf life of ODT medications. The frequency of HAE attacks requiring treatment during active LTP therapy was summarized descriptively as attack rates per person-year, together with corresponding 95% confidence intervals (CIs) based on exact Poisson confidence limits. In addition, the Kaplan-Meier method was applied to assess time to the first HAE attack requiring treatment, with follow-up starting at LTP initiation. Censoring was applied at discontinuation of LTP, defined as an interruption of more than 90 days without any LTP prescription, death, or end of the study period.

In contrast, the MCR dataset provided detailed information on all HAE attacks, including both treated and untreated episodes. Attack frequency was recorded as reported by treating physicians based on patient documentation and clinical assessments. The MCR dataset also included additional clinical details, such as the location of attacks, allowing for a more comprehensive assessment of disease burden beyond what was available in the claims data. To assess the plausibility of the claims-based attack identification algorithm, physician-documented treated attack frequencies were used as an external clinical benchmark and compared with prescription-based attack estimates. Overall, prescription-based attack estimates showed reasonable agreement with physician-documented treated attack frequencies and supported the plausibility of ODT-based attack identification in claims data.

#### HCRU

Information on HCRU from the initiation of LTP onward was available only for the claims data cohorts (AOK PLUS and GWQ) and was collected during the patient-individual follow-up period (including index date). A distinction was made between all-cause and HAE related cases. A case was defined to be HAE related if the associated primary diagnosis was D84.1 or T78.3. Outcomes for all-cause and HAE related HCRU consisted of the number of hospitalizations and sick leave days absent from work, the latter being available exclusively for the AOK PLUS cohort. The number of outpatient physician contacts, available only for the GWQ cohort, was additionally assessed and stratified by physician type, including general practitioners (GPs), specialized pediatricians, dermatologists, and otolaryngologists (ENT specialists). Outpatient contacts were quantified based on the number of reimbursed outpatient services documented in SHI records during the observation period. All HCRU outcomes were assessed over the entire available observation period and at predefined time intervals of 1, 2, 3, 4, and 5 years following initiation of LTP. All HCRU outcomes were summarized descriptively. Hospitalizations and outpatient visits were reported as rates per person-year (events/PY) with corresponding 95% CIs based on exact Poisson confidence limits, as well as mean numbers of events per patient and observed ranges. Sick leave outcomes were reported as proportions of patients with at least 1 sick leave episode, mean sick leave days, and ranges.

#### Healthcare costs

Healthcare costs from the payor-perspective included direct costs for inpatient care (ie, hospitalizations), outpatient care, and medication. Inpatient hospitalization cost refers to the total expenses incurred for medical services provided during a hospital stay. These costs included all aspects of inpatient care, such as room and board, medical procedures, nursing care, medications, and diagnostic tests. Outpatient cost referred to the total expenses incurred for medical services provided during outpatient visits. They were determined based on the billing data from the Associations of Statutory Health Insurance Physicians for GPs and various specialist groups. Outpatient drug prescriptions were valued based on the gross pharmacy retail price at the respective date of dispensation, excluding any mandatory or voluntary discounts as well as co-payments from patients. Costs were assessed from the SHI perspective and reported in Euros per person-year to account for differences in follow-up duration between patients. For each patient, total accrued costs during the observation period were divided by the corresponding follow-up time in person-years, and summary statistics were calculated across the study population.

## Results

### Patient baseline characteristics

Based on the predefined selection criteria, 77 patients with HAE who received at least 1 LTP therapy were identified in the 2 German claims databases: 41 from AOK PLUS and 36 from GWQ (Fig. 1). The median follow-up time was 4.3 years for AOK PLUS-insured patients and 4.5 years for those insured by GWQ. The mean follow-up per patient was 3.4 years (AOK PLUS) and 3.5 years (GWQ), corresponding to a total of 138.3 and 129.2 person-years in each claims database, respectively. Additionally, 20 patients were included in the MCR cohort with a fixed follow-up period of 1 year per patient.

**Fig. 1:**
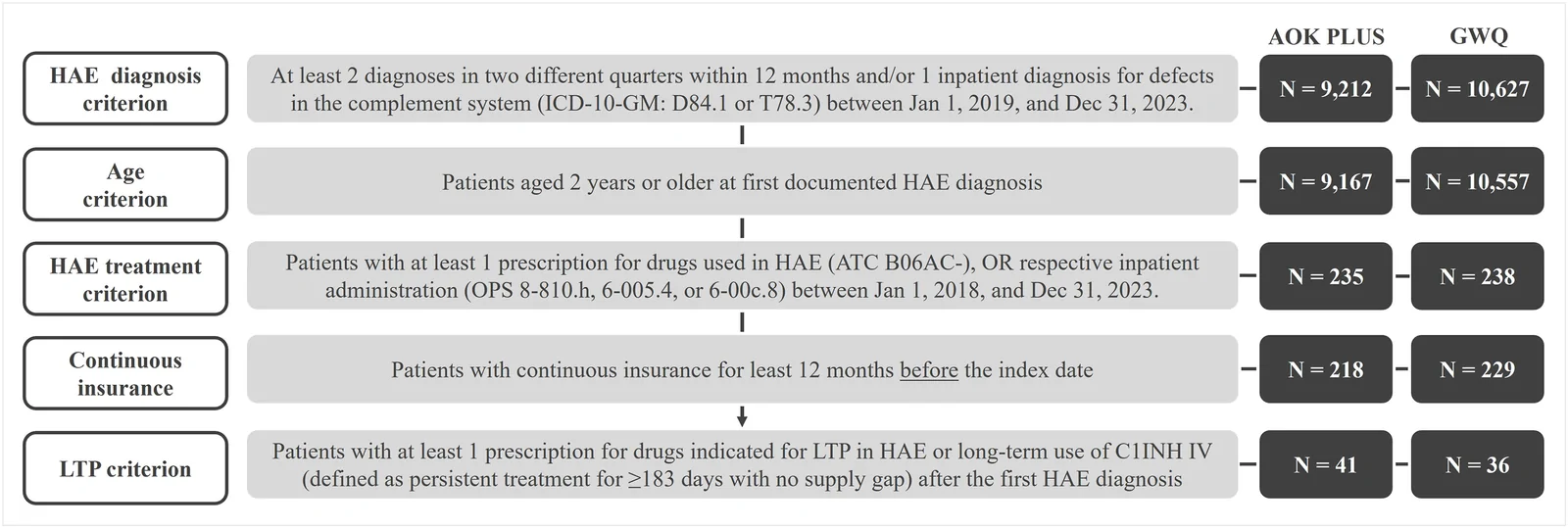
Attrition Chart. **Abbreviations:** C1INH, C1-Esterase inhibitors; HAE, hereditary angioedema; ICD-10-GM, International Classification of Diseases - German Modification; IV, intravenous LTP, long-term prophylaxis.

Table 1 summarizes the baseline characteristics of patients with HAE across the AOK PLUS, GWQ, and MCR cohorts, including demographics, comorbidities, and prior HAE treatments. The mean age ranged from 40.0 years (MCR) to 44.6 years (GWQ). Prior to LTP treatment, plasma-derived C1INH was the most frequently used ODT for acute HAE attacks, followed by icatibant. The MCR cohort included patients with different HAE subtypes, most commonly HAE with C1INH deficiency, accounting for 85.0% of cases, and HAE with normal C1INH, comprising 15.0%. Only patients with HAE with normal C1INH and a genetically confirmed mutation in one of the genes known to cause this subtype were included. The mean disease duration at LTP start was 16.7 years (SD: 13.2), with a median of 14.6 years (range: 2–47 years), indicating substantial variability in disease history among patients.

### Treatment patterns

The distribution of initiated LTP regimens varied across study cohorts when considering all LTP regimens started during the observation period. Because patients could initiate more than 1 LTP regimen over time, they may contribute multiple observations. In the claims data cohorts (AOK PLUS and GWQ), lanadelumab was the most frequently initiated LTP therapy (57.1%), followed by C1INH IV (39.0%), C1INH SC (28.6%), and berotralstat (14.3%). In contrast, nearly all patients in the MCR cohort initiated lanadelumab (85.0%), reflecting treatment patterns observed in the targeted specialized HAE care center.

In the claims data cohorts, time from initiation of the first LTP regimen to another LTP regimen was depicted using Kaplan–Meier survival analysis (Fig. 2). In the AOK PLUS cohort, 18 patients (44%) switched their initial LTP regimen during follow-up, with the 25th percentile for time to switch reached after approximately 2.6 years and the median time to switch after 3.9 years. In the GWQ cohort, 9 patients (25%) switched therapies, with the 25th percentile reached after approximately 3.1 years. Owing to the limited number of switching events, the median time to switch could not be estimated for the GWQ cohort.

**Fig. 2:**
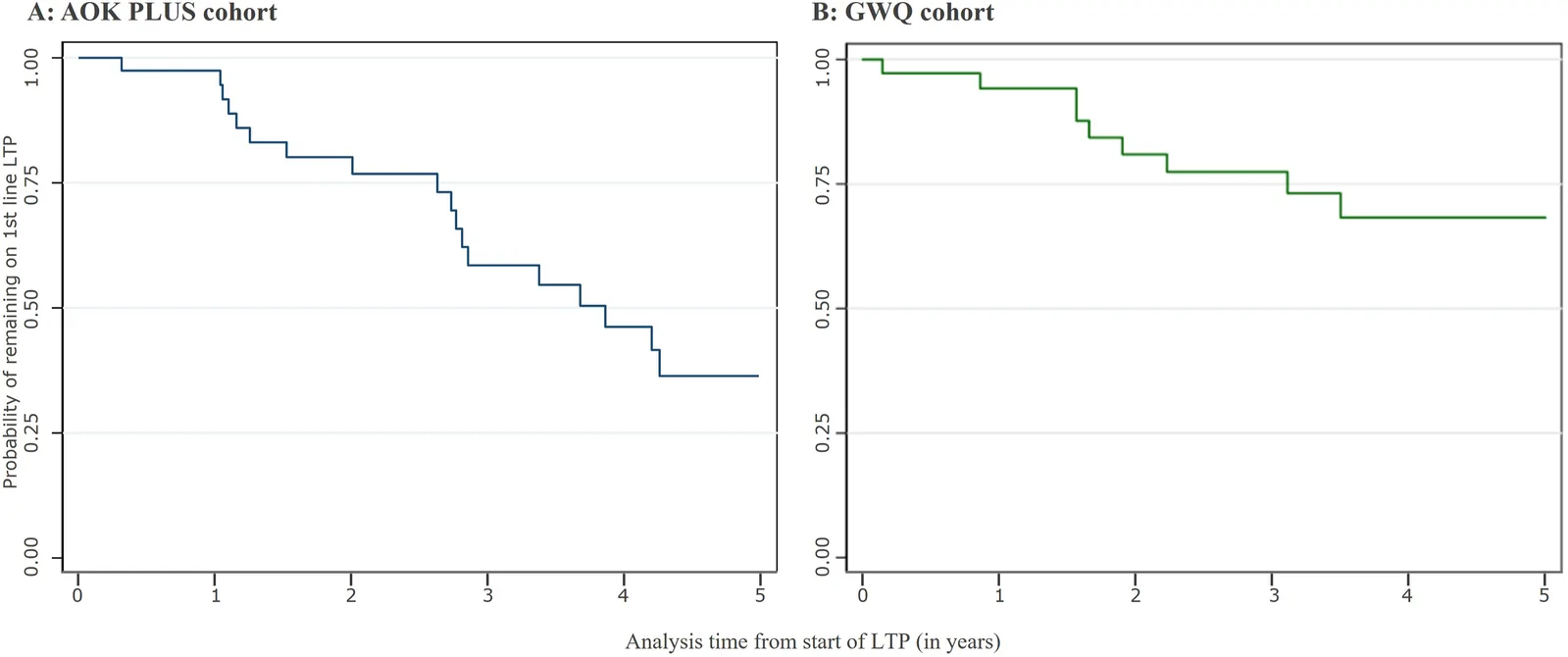
Kaplan–Meier curves for time to switch from the initiated LTP regimen among patients with HAE in the claims data cohorts: (A) AOK PLUS and (B) GWQ. **Abbreviations:** HAE, hereditary angioedema; LTP, long-term prophylaxis.

Treatment sequences derived from the consolidated claims data cohorts (AOK PLUS and GWQ) are summarized in Fig. 3. Among 77 patients initiating LTP, C1INH IV was the most common first-line (1 L) regimen (39.0%), followed by lanadelumab (31.2%), C1INH SC (20.8%), and berotralstat (9.1%). Overall, 27 patients (35.1%) progressed to a 2 L regimen, while 50 (64.9%) remained on their 1 L treatment during the observation window. Among 27 patients who advanced to 2 L, the predominant destination was lanadelumab (70.4%), followed by C1INH SC (18.5%) and berotralstat (11.1%); C1INH IV was not observed as a 2 L regimen. Progression beyond 2 L was infrequent; only 7.4% transitioned to a third-line (3 L) regimen. Observed 3 L destinations were lanadelumab and C1INH SC.

**Fig. 3:**
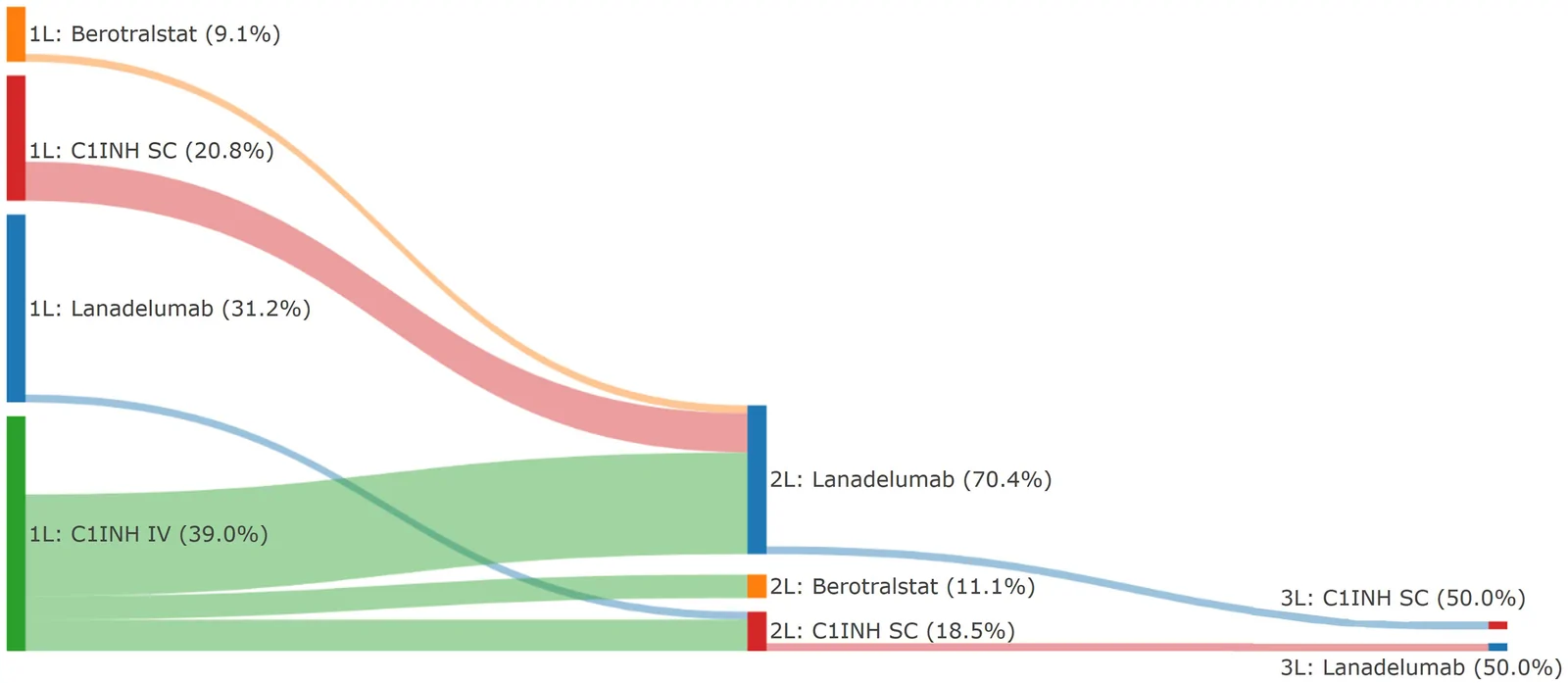
Sequential Switching Patterns Between LTP Therapies in HAE. **Abbreviations:** C1INH, C1-Esterase inhibitors; HAE, hereditary angioedema; IV, intravenous; LTP, long-term prophylaxis; SC, subcutaneous.

Among the lanadelumab starters, intermittent dosing, defined as alternating between bi-weekly and four-weekly usage (197–335 DDD/year), was recorded in 33% of AOK PLUS and 57% of GWQ patients (90-day grace period). As part of a sensitivity analysis, the impact of different grace periods on dosing classifications was assessed. With a 365-day grace period, the proportion remained stable at 33% in AOK PLUS but increased to 61% in GWQ. No patient adhered strictly to an every 4-week regimen (168–196 DDD/year). However, a subset of patients received cumulative doses below this expected range (<168 DDD/year). While no patients fell into this category under the 90-day grace period, 24% in AOK PLUS and 22% in GWQ were classified as such under the 365-day grace period, suggesting sporadic or irregular use.

### HAE attack frequency

Among patients receiving any type of LTP, 36% (N = 77) remained attack-free throughout the first year of treatment (Fig. 4). In the first year following LTP initiation, the total follow-up time was 30.8 person-years in AOK PLUS and 29.1 person-years in GWQ. The estimated rates for treated HAE attacks per person-year were 1.6 (95%-CI: 1.2–2.1) and 2.0 (95%-CI: 1.5–2.5), respectively, with a median of 1 attack per patient (range: 0–5 in AOK PLUS; 0–8 in GWQ). By the second year, total follow-up decreased to 11.9 person-years in AOK PLUS and 15.4 person-years in GWQ, with lower estimated attack rates per person-year of 1.2 (95%-CI: 0.6–2.0) and 1.6, (95%-CI: 1.0–2.3) respectively, suggesting a decline in attack frequency over time. Over the total follow-up period, the cumulative observation time was 45.9 person-years in AOK PLUS and 56.5 person-years in GWQ, with overall attack rates of 1.4 (95%-CI: 1.1–1.8) and 2.0 (95%-CI: 1.7–2.4) per person-year, respectively. The median number of attacks per patient remained low at 1 in AOK PLUS (range: 0–9) and 2 in GWQ (range: 0–25). Furthermore, time-to-event analysis showed that the median time from LTP initiation to the first HAE attack requiring treatment was 114 days (25th percentile: 1 day) in AOK PLUS and 132 days (25th percentile: 1 day) in GWQ. Notably, 32% and 30% of the observed attacks in the first follow-up year were recorded at day 1 after LTP initiation.

**Fig. 4:**
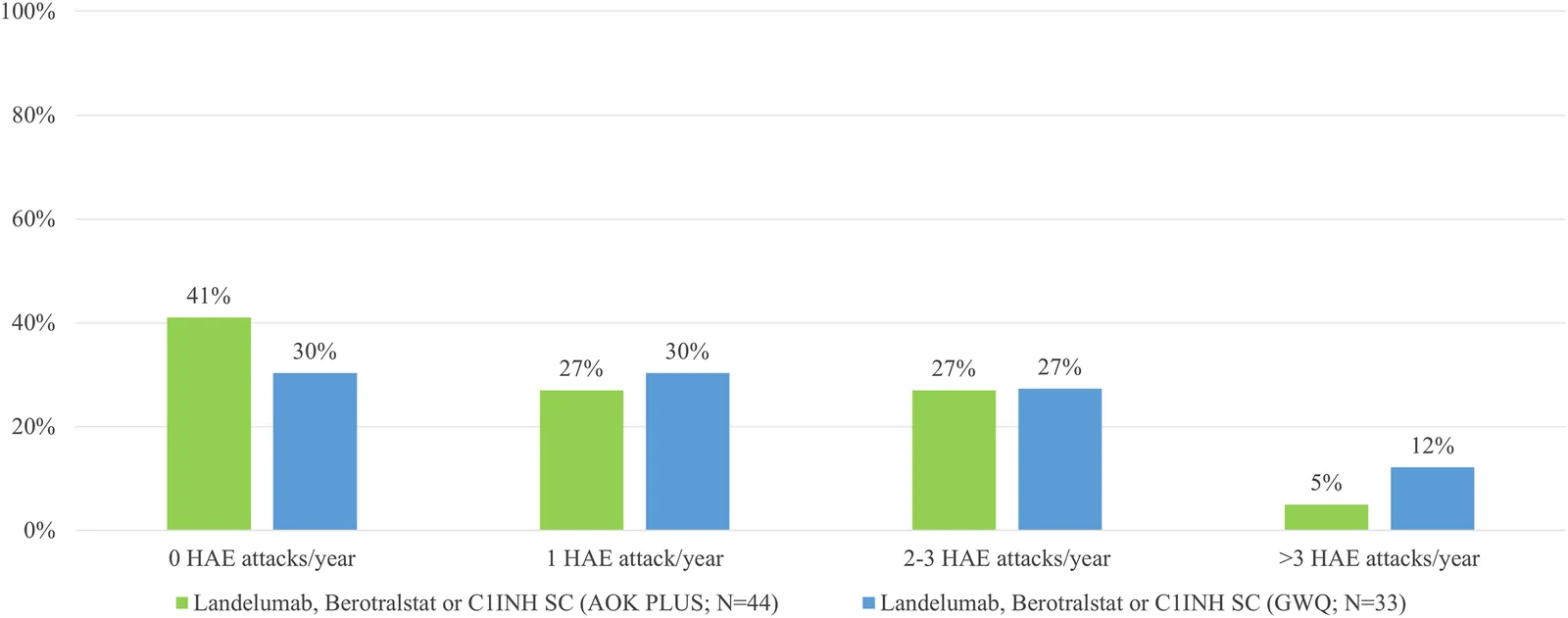
Distribution of HAE attacks requiring treatment in the first year since start of LTP therapy; analysis based on claims data. **Abbreviations:** HAE, hereditary angioedema; LTP, long-term prophylaxis; SC, subcutaneous.

A comparison of retrospective ODT prescription data with chart-documented HAE attacks requiring treatment indicated overall agreement between the 2 approaches, with some residual discordance: In 35% of cases, prescription-based counts were higher than chart-documented treated attacks, and in 10% of cases they were lower. The proportion of patients classified as attack-free was notably higher based on medical chart documentation (80%) compared to prescription data for ODT (45%), while the proportion of patients experiencing at least 1 attack was lower (20% vs. 55%; Figure S 1). This difference may partly reflect misclassification, as some ODT dispensing may have been used for short-term prophylaxis or (re)stocking of on-demand medication rather than acute attack treatment.

Analysis of attack location and treatment necessity showed a total of 25 HAE attacks among 20 patients in the MCR sample, with an average of 1.25 attacks per patient. The most common attack locations were abdominal (44%) and peripheral (32%), followed by facial (20%) and unknown sites (8%). Among 13 treated attacks, 54% were abdominal, 38% peripheral, 8% facial, and 8% of unknown location. The mean number of treated attacks per patient was 0.65, indicating that a substantial portion of attacks (12/25) did not require acute treatment.

### HCRU

Inpatient HCRU among patients with HAE starting LTP was analyzed for both claims datasets over a period of up to 5 years, based on a mean follow-up time of 3.4 years for AOK PLUS and 3.5 years for GWQ (Table 2). During the total follow-up period, HAE-related hospitalizations were recorded in 22% of patients (AOK PLUS) and 41.7% (GWQ), with an event rate of 0.10 (95%-CI: 0.06–0.17) and 0.21 (95%-CI: 0.14–0.30) per person-year, respectively. For all-cause hospitalizations, a higher proportion of patients required inpatient care, with 31.7% (AOK PLUS) and 50.0% (GWQ) having at least 1 hospitalization. The event rate was 0.23 (95%-CI: 0.16–0.33; AOK PLUS) and 0.43 (95%-CI: 0.32–0.56; GWQ) per person-year. Across both datasets, HAE-related hospitalization rates declined over time, with the highest rates observed in the first 2 years following LTP initiation.

**Table 2:** HCRU subsequent to start of LTP based on AOK PLUS and GWQ claims (Inpatient Care).

	1st year		2nd year		3rd year		4th year		5th year		Total follow-up	
Sample	**AOK PLUS**	**GWQ**	**AOK PLUS**	**GWQ**	**AOK PLUS**	**GWQ**	**AOK PLUS**	**GWQ**	**AOK PLUS**	**GWQ**	**AOK PLUS**	**GWQ**
N Patients	41	36	34	32	30	29	27	23	22	20	41	36
**Length of follow-up in person-years**												
Total sum	37.5	34.8	32.5	30.2	27.8	25.6	24.5	21.2	16.0	13.9	138.3	125.8
**HAE-related hospitalizations [diagnosed cases with ICD-10-GM: D84.1 or T78.3]**												
Events/PY	0.08	0.29	0.15	0.20	0.11	0.12	0.08	0.14	0.06	0.29	0.10	0.21
95%-CI	0.02–0.23	0.14–0.53	0.05–0.36	0.07–0.43	0.02–0.32	0.02–0.34	0.01–0.29	0.03–0.41	0.01–0.35	0.08–0.74	0.06–0.17	0.14–0.30
Mean	0.07	0.28	0.15	0.19	0.10	0.10	0.07	0.13	0.05	0.20	0.34	0.72
Range	0–1	0–3	0–2	0–3	0–2	0–1	0–2	0–2	0–1	0–3	0–4	0–4
**All-cause hospitalizations**												
Events/PY	0.19	0.46	0.25	0.53	0.14	0.23	0.37	0.38	0.31	0.65	0.23	0.43
95%-CI	0.07–0.38	0.26–0.75	0.11–0.49	0.30–0.86	0.04–0.37	0.09–0.51	0.17–0.70	0.16–0.74	0.10–0.73	0.30–1.23	0.16–0.33	0.32–0.56
Mean	0.17	0.44	0.24	0.50	0.13	0.21	0.33	0.35	0.23	0.45	0.78	1.50
Range	0–2	0–3	0–2	0–7	0–2	0–1	0–5	0–4	0–2	0–6	0–5	0–11

**Abbreviations:** CI, Confidence Interval; HAE, hereditary angioedema; HCRU, Healthcare Resource Utilization; ICD-10-GM, International Classification of Diseases - German Modification; LTP, long-term prophylaxis; PY, Person-year

Outpatient HCRU was assessed based on GWQ claims data for patients initiating LTP, with a mean follow-up duration of 3.5 years (Table 3). During the total observation period, 94.4% of patients had at least 1 reimbursed visit to a GP, corresponding to an event rate of 13.27 (95%-CI: 12.64–13.92) visits per person-year. GP visits accounted for the majority of outpatient encounters and remained consistently frequent over time, with event rates ranging from 11.71 to 18.89 visits per person-year across follow-up years. Contacts with pediatric specialists were less frequent, with 22.2% of patients having at least 1 visit and an overall event rate of 1.12 (95%-CI: 0.94–1.32) visits per person-year, showing a slight decline over time. Visits to dermatologists and otolaryngologists were documented for 47.2% of patients each, with comparable event rates of 0.62 (95%-CI: 0.49–0.77) and 0.60 (95%-CI: 0.48–0.78) visits per person-year for both specialties, peaking in the first 2 years and decreasing thereafter. Overall, outpatient visit frequencies showed modest year-to-year variation without a consistent increasing or decreasing trend.

**Table 3:** HCRU subsequent to start of LTP based on GWQ claims (Outpatient Care).

	1st year	2nd year	3rd year	4th year	5th year	Total follow-up
N Patients	36	32	29	23	20	36
**Length of follow-up in person-years**						
Total sum	34.8	30.2	25.6	21.2	13.9	125.8
**GPs visits**						
Events/PY	14.78	14.92	11.71	13.13	18.89	13.27
95%-CI	13.53–16.12	13.57–16.36	10.42–13.11	11.63–14.76	16.67–21.32	12.64–13.92
Mean	14.31	17.46	10.34	12.09	13.10	46.36
Range	0–103	0–81	1–46	0–60	0–52	0–197
**Pediatric specialists**						
Events/PY	1.58	1.22	0.94	0.57	0.94	1.12
95%-CI	1.19–2.05	0.86–1.69	0.60–1.39	0.29–0.99	0.50–1.60	0.94–1.32
Mean	1.53	1.16	0.83	0.52	0.65	3.92
Range	0–10	0–9	0–7	0–4	0–6	0–36
**Dermatologists**						
Events/PY	0.49	1.12	0.62	0.28	0.36	0.62
95%-CI	0.28–0.78	0.78–1.57	0.36–1.01	0.10–0.62	0.12–0.84	0.49–0.77
Mean	0.47	1.06	0.55	0.26	0.25	2.17
Range	0–5	0–23	0–3	0–5	0–3	0–26
**Otolaryngologists**						
Events/PY	0.63	0.79	0.51	0.47	0.50	0.60
95%-CI	0.40–0.96	0.51–1.18	0.27–0.87	0.23–0.87	0.20–1.04	0.48–0.76
Mean	0.61	0.75	0.45	0.43	0.35	2.11
Range	0–6	0–8	0–5	0–5	0–4	0–16

**Abbreviations:** CI, Confidence Interval; GP, General practitioner; HAE, hereditary angioedema; HCRU, Healthcare Resource Utilization; LTP, long-term prophylaxis; PY, Person-year

Sick leave data were available for patients in the AOK PLUS dataset (Table 4). During the total follow-up period, 43.9% of patients had at least 1 HAE-related sick leave episode. Across the overall study population, the mean duration of HAE-related sick leave was 34.9 days. For all-cause sick leave, the proportion of affected patients was higher, with 65.9% expe-riencing at least 1 episode. The mean duration of an all-cause sick leave was 83.5 days (median: 24 days).

**Table 4:** HCRU subsequent to start of LTP based on AOK PLUS claims (Sick leave days).

	1st year	2nd year	3rd year	4th year	5th year	Total follow-up
N Patients	41	34	30	27	22	41
Length of follow-up [in person-years]						
Total sum	37.5	32.5	27.8	24.5	16.0	138.3
HAE-related sick leave days [diagnosed cases with ICD-10-GM: D84.1 or T78.3]						
% With ≥1 leave	26.8%	26.5%	20.0%	22.2%	18.2%	43.9%
Mean	5.7	9.4	10.6	11.5	11.3	34.9
Range	0–180	0–271	0–275	0–196	0–234	0–922
All-cause sick leave days						
% With ≥1 leave	53.7%	52.9%	53.3%	63.0%	45.5%	65.9%
Mean	15.0	18.2	30.0	29.2	22.7	83.5
Range	0–180	0–276	0–275	0–196	0–234	0–927

**Abbreviations:** HAE, hereditary angioedema; HCRU, Healthcare Resource Utilization; ICD-10-GM, International Classification of Diseases - German Modification; LTP, long-term prophylaxis; PY, Person-year

### Healthcare costs

The total direct healthcare costs per person-year varied between the AOK PLUS and GWQ cohorts, with medication costs for outpatient pharmacy dispensations being the primary cost driver in both datasets (Fig. S 2). The average annual cost per person-year for outpatient medications was €233,190 (AOK PLUS) and €206,392 (GWQ), accounting for the majority of total expenditures. Inpatient care costs were relatively low in AOK PLUS, with an average of €702 per person-year, while costs were substantially higher in the GWQ dataset at €6140 per person-year. This marked difference was attributable to both a greater frequency of hospitalizations per patient and higher average costs per admission (€14,299 in GWQ vs. €3032 in AOK PLUS). In addition, the variability in inpatient costs per case differed considerably between datasets: while costs in the AOK PLUS sample ranged from €0 to €21,809, the range in the GWQ sample extended from €0 to €558,250, indicating the presence of a small number of exceptionally high-cost cases that likely contributed disproportionately to the overall cost burden in the GWQ sample. Outpatient care costs were similar across both cohorts, amounting to €1027 (AOK PLUS) and €1005 (GWQ) per person-year.

## Discussion

This is the first study that combines German SHI claims data with physician chart review data to evaluate treatment patterns, frequency of attacks requiring ODT, HCRU, and costs associated with LTP in HAE. By leveraging complementary data sources, the study provides insights into both HCRU and clinical disease burden that would not be fully captured using either data source alone. Across data sources, patients initiating LTP were predominantly middle-aged adults with long-standing disease histories and diverse comorbidity profiles.

Lanadelumab was the most frequently initiated prophylactic therapy, particularly in the MCR cohort, whereas C1INH prophylaxis showed distinct patterns by route of administration: Use of C1INH IV declined over time, whereas C1INH SC remained comparatively stable and was more commonly prescribed to patients switching from other regimens. Overall, most patients remained on their initial LTP regimen, while around one-third switched therapies. When switching occurred, transitions were predominantly toward SC or oral prophylaxis options, consistent with a gradual shift away from IV administration towards more convenient routes. Recent European real-world data confirm these trends, indicating ongoing use of plasma-derived C1INH SC for LTP, including switching from IV to SC administration, alongside increasing uptake of newer options such as lanadelumab.^14^^,^^15^ For instance, a German prescription analysis conducted between 2018 and 2020 demonstrated that, following the introduction of lanadelumab, a substantial number of patients transitioned from established LTP regimens to lanadelumab.^16^ Moreover, expert surveys in Germany noted that only about one-quarter of patients with HAE were on any prophylaxis in the pre-lanadelumab era, but this rate is expected to rise with the availability of modern agents.^24^ Thus, while lanadelumab currently dominates new prophylactic initiations in specialized cohorts, the continued use of C1INH concentrates in first-line settings underscores its established place in HAE management. At the same time, continued use of C1INH is expected in specific patient groups, as it is generally preferred for women planning pregnancy and for pregnant or breastfeeding women.^5^ In addition, our analysis identified considerable variability in real-world lanadelumab dosing patterns, including intermittent use and cumulative dosing frequencies below those expected for standard bi-weekly or four-weekly regimens. These findings may reflect individualized treatment strategies, dose interval extension in well-controlled patients, adherence-related factors, or differences in disease severity. Such variability may also contribute to residual disease activity and should be considered when interpreting breakthrough attacks observed during follow-up. Reasons for treatment switching could not be directly assessed within the available datasets. Potential contributing factors may include patient preference, route of administration, treatment burden, perceived effectiveness, physician decision-making, and evolving treatment availability over time. The continued use of plasma-derived C1INH alongside newer targeted therapies indicates that multiple LTP options remain clinically relevant and may address different patient needs throughout the patient journey. Although the underlying reasons for treatment switching could not be assessed in the available data sources, the observed transition from IV prophylaxis toward SC and oral treatment options may reflect several factors reported in clinical practice, including reduced treatment burden and improved convenience for patients. However, factors such as comparative effectiveness, tolerability, physician preference, reimbursement considerations, therapy adherence, and individual patient circumstances and preferences may also have contributed to switching decisions and could not be evaluated in this study.

In this claims data analysis, a substantial proportion of patients had at least 1 treated HAE attack recorded within the first year after LTP initiation. Persistent attacks despite LTP likely reflect heterogeneity in disease severity, differences in individual treatment response, variability in treatment adherence, individualized dosing strategies, and the occurrence of attacks during the early period following treatment initiation. Attacks recorded early after LTP initiation may be attributable to therapy initiation during periods of high disease activity, residual attacks before full prophylactic effect or steady-state exposure is achieved, or delayed refilling of on-demand medication following attacks that prompted LTP initiation, rather than representing true breakthrough attacks under established prophylaxis. Clinical trial and observational evidence consistently demonstrate rapid and sustained attack control with contemporary LTP agents.^25^, ^26^, ^27^, ^28^, ^29^ Time-to-event analysis revealed that many patients experienced prolonged protection after starting LTP, while a subset of patients required ODT as early as day 1. Although these early events should be interpreted with caution, they indicate that attack risk may persist during the early treatment period and that complete disease control may not be achieved shortly after treatment initiation. Furthermore, our observations are consistent with other recent real-world studies, emphasizing the variability in individual response to LTP and the need for ongoing monitoring and treatment adjustments ^25^^,^^26^. From a clinical perspective, these results highlight that initiation of LTP may not necessarily be expected to result in early and complete elimination of attack risk. Continued access to ODT and close follow-up during the early LTP period remain important, particularly for patients initiating prophylaxis during periods of increased disease activity.

Prescription data for ODT are frequently used as proxies for the frequency of treated HAE attack in real-world studies, yet their accuracy in reflecting true disease burden has been debated.^30^, ^31^, ^32^, ^33^, ^34^ Our findings support the view that prescription records of ODT offer a practical, albeit imperfect, surrogate for the frequency of treated HAE attacks. Validation research has shown moderate consistency between self-reported and prescription-based medication use, while studies in HAE populations highlight that a substantial proportion of attacks remain untreated due to mild severity or patient preference ^34^^,^^35^. This underscores the need for careful interpretation of dispensing data of ODT, especially when used to evaluate treatment effectiveness or disease activity. Given the variability in attack severity and individual thresholds for initiating treatment, real-world assessments should consider integrating multiple data sources, such as clinical records or patient-reported outcomes, to ensure a more comprehensive understanding of HAE burden and management. However, each data source has inherent limitations. In this context, our MCR was conducted at a single specialized center and should therefore be interpreted as a complementary perspective rather than as a definitive benchmark. Despite its limitations, the observed consistency between prescription-based and physician-documented treated attack frequencies supports the use of ODT prescription claims as a pragmatic outcome measure in real-world database studies, particularly when detailed clinical information is unavailable.

Our analysis of German SHI claims data indicated that a considerable proportion of patients with HAE undergoing LTP continued to require inpatient care, with between one-fifth and nearly half of patients experiencing at least 1 HAE-related hospitalization during the observation period. These results are consistent with broader findings on the burden of HAE reported in multinational and systematic reviews. For example, in a recent multinational patient survey nearly half of the patients with HAE reported emergency or hospital-based care within a 6-month period, reflecting significant ongoing morbidity.^36^ Similarly, a recent systematic review emphasized that hospitalization rates remain substantial across real-world cohorts, particularly in patients with suboptimal disease control.^37^ These findings point to variability in response and disease trajectory, reinforcing the need for close monitoring in patients on LTP. Furthermore, our analysis showed consistently high outpatient care utilization among patients receiving LTP, with nearly all patients visiting GPs regularly and moderate use of specialist services. This pattern aligns with prior real-world studies indicating that regular medical follow-up remains essential in LTP-treated patients to manage comorbidities, monitor treatment response, and adjust therapy as needed.^38^ Importantly, the persistence of hospitalizations and ongoing outpatient care among patients receiving LTP suggests that substantial healthcare needs remain even after LTP initiation. These findings indicate that successful HAE management extends beyond attack prevention alone and requires ongoing monitoring, treatment optimization, and management of individual patient needs.^39^

Beyond hospitalizations and outpatient visits, the substantial proportion of patients requiring HAE-related sick leave, affecting nearly half of the patients in our study, underscores the broader socioeconomic burden of the disease, particularly its impact on daily functioning and work productivity. This substantial impairment that was observed despite prophylactic treatment further suggests that treatment success should not be evaluated solely based on reduction of attack frequency but should also consider broader patient-centered outcomes, as highlighted in current HAE clinical guidelines.^5^^,^^7^ Furthermore, work absences due to HAE attacks contribute significantly to the indirect cost burden, a trend also noted in prior studies assessing the humanistic burden of illness and productivity losses associated with HAE.^36^^,^^40^ In our claims-based analysis, however, sick-leave days and hospitalizations could not be definitively attributed to acute HAE attacks because the underlying reason is not consistently captured in diagnosis coding and detailed clinical context is unavailable.

Building on these considerations, our cost analysis highlights the substantial economic burden associated with HAE prophylaxis in the SHI setting. The present analysis focused on healthcare costs observed after initiation of LTP and was not designed to evaluate changes in healthcare expenditures before versus after prophylaxis initiation. Future studies should assess longitudinal cost trajectories to better quantify the economic impact of modern prophylactic treatment strategies. Outpatient medication expenses emerged as the primary cost driver, while inpatient and outpatient care contributed a comparatively minor share. These findings are consistent with previous European studies demonstrating that LTP therapies account for the majority of direct healthcare costs. For instance, a recent Dutch study reported that medication-related expenditures constituted the largest proportion of total costs in patients with active disease,^41^ and a global systematic review confirmed that pharmacologic prophylaxis remains the predominant contributor to overall HAE-related spending.^37^ However, while medication costs represented the largest share of direct healthcare expenditures, these findings should be interpreted in the context of the substantial clinical burden associated with HAE. Effective prophylaxis may increase pharmaceutical expenditures while potentially reducing attack-related HCRU and productivity losses. Consequently, comprehensive assessments of value should consider both the costs of prophylactic treatment and its potential impact on disease burden, subsequent HCRU, and indirect costs from a societal perspective.

### Limitations

This study has several important limitations inherent to the use of retrospective real-world data. First, the lack of HAE-specific ICD-10 codes in German SHI claims data may result in misclassification, as patients with other forms of angioedema or related conditions could be inadvertently included or excluded. However, the validity of patient identification is supported by the high proportion of patients with a documented history of HAE-specific treatment prior to LTP initiation. In the claims cohorts, approximately 80% of patients had received plasma-derived C1INH and nearly half had received icatibant during the baseline period, consistent with established treatment patterns for confirmed HAE and supporting the specificity of the applied identification algorithm. While applying treatment-based inclusion criteria improves patient identification, it may still misclassify individuals who receive HAE therapies for diagnostic testing, off-label use, or prophylaxis without a confirmed diagnosis, and it may exclude untreated patients with confirmed HAE. In addition, selection bias cannot be excluded. By restricting the analysis to patients who initiated LTP, the study population may differ systematically from patients with HAE who were untreated or managed exclusively with ODT. Furthermore, patients receiving different LTP regimens may differ in disease severity, treatment history, comorbidity burden, or treatment preferences, factors that were not fully observable in the available data sources. Second, while prescription data for ODT provide a practical proxy for estimating attack frequency, they may not fully reflect actual treatment needs. Medications may be dispensed to refill emergency supply but remain unused. According to guideline recommendations, patients should maintain sufficient supply to treat at least 2 attacks and carry on-demand therapy at all times.^5^, ^6^, ^7^ In addition, because claims data do not capture therapeutic intent, some dispensing classified as ODT may in fact represent short-term prophylaxis (eg, peri-procedural use), which could lead to misclassification of prophylactic use as treated attacks. Results should therefore be interpreted with appropriate caution. Conversely, retrospective chart reviews may underreport attacks requiring ODT if they were self-managed and not communicated during follow-up visits. In addition, as chart review data are typically collected at a single site, they might not capture attacks treated at other healthcare facilities. As a result, both data sources have inherent limitations: prescription records may capture intention to treat rather than actual use, while chart data may miss events that did not prompt clinical action. This underscores the importance of triangulating multiple data sources to better understand real-world disease activity. Third, claims data inherently lack clinical detail. Information on symptom severity, physician decision-making, subtype classification, or patient-reported outcomes is not captured. This limits the ability to fully evaluate disease burden, treatment rationale, or quality-of-life impact. Similarly, data on sick leave include only certified absences and may underestimate shorter or informal work disruptions, such as presenteeism, related to HAE. Fourth, while the MCR provided detailed clinical information beyond what was available in claims data, some variables, including family history, laboratory parameters, disease severity measures, and adverse events, were not consistently documented in the retrospective medical records and therefore could not be systematically assessed. In addition, the single-center nature of the MCR may limit the completeness of certain clinical characteristics and treatment history. Fifth, because this study was designed as a descriptive assessment of treatment patterns, related outcomes, HCRU, and costs, it did not include adjusted comparative analyses between individual LTPs. Consequently, observed differences between treatment groups should not be interpreted as evidence of comparative effectiveness, as important confounding factors such as disease severity, and physician prescribing preferences could not be fully accounted for. Sixth, cost analyses are based on gross pharmacy retail prices, and do not account for mandatory rebates (eg, statutory manufacturer discounts and fixed pharmacy margins) or confidential negotiated discounts between manufacturers and insurers. As such, the reported expenditures likely overestimate the true financial burden to SHI funds. Seventh, given that HAE is a hereditary disorder, it is not uncommon for multiple family members to be affected. In some cases, medication may be shared within families, especially in emergency situations or when 1 member has surplus supply. This informal redistribution of medication is not captured in claims data and may distort utilization patterns. Finally, the generalizability of the findings may be limited. The claims analyses were based on 2 German SHI cohorts and may not fully reflect treatment patterns in privately insured patients or healthcare systems outside Germany. Furthermore, the MCR was conducted at a single specialized HAE center and may not be representative of all treatment settings or patient populations in Germany.

## Conclusion

This study provides comprehensive real-world evidence on the routine use of LTP among patients with HAE in Germany by leveraging complementary insights from SHI claims data and physician chart review data. Although currently available LTP therapies were associated with prolonged periods without treated attacks in many patients, a substantial proportion of patients continued to experience breakthrough attacks, require hospitalizations, and incur considerable healthcare costs, highlighting the ongoing burden of HAE in routine clinical practice. Despite guideline recommendations aiming for complete disease control and life normalization, the claims-based analysis showed that the majority of patients experienced HAE attacks within the first year after LTP initiation, with only a minority remaining attack-free. While attack rates declined over time and time-to-event analyses suggest that some patients benefit from prolonged protection, the frequent occurrence of early breakthrough episodes highlights the need for optimized LTP strategies and individualized treatment monitoring. Furthermore, the study demonstrates that prescription records for ODT can serve as a pragmatic proxy for treated HAE attacks in claims-based analyses, while emphasizing the importance of complementary clinical data for a comprehensive assessment of disease burden. Future research should further evaluate factors associated with residual disease burden, treatment switching, and comparative outcomes across available prophylactic therapies to support evidence-based treatment decision-making and healthcare planning. In addition, subsequent studies should explore whether and to what extent the higher treatment costs associated with LTP are offset by reductions in attack-related healthcare costs, thereby supporting evidence-based treatment decision-making and public healthcare planning.

## Availability of data and materials

The data analyzed in this study are not publicly available due to the data protection regulations of the German Social Code Book (‘Fünftes Sozialgesetzbuch’, SGB V).

## Ethics statement

This study was conducted in accordance with the ethical principles of the current Declaration of Helsinki, is consistent with applicable regulatory requirements, and was approved by the Ethics Committee of the University of Ulm (reference number: 453/24), Ulm, in Germany in accordance with § 15 of the Professional Code of Conduct of the State Medical Association of the federal state Baden-Württemberg. The study protocol was reviewed and approved by members of the scientific steering committee, including the study sponsor, the claims database holders (AOK PLUS and GWQ ServicePlus AG), the research organization conducting the analysis (GIPAM GmbH), the local principal investigator, and the authors. Explicit informed consent to access electronic medical health records and claims data was not required for this study, as the patients’ privacy was guaranteed and the data was completely anonymized.

## Submission declaration

All authors have approved the submission of this manuscript. The results have not been previously published.

## Statement regarding the use of generative AI and AI-assisted technologies

Nothing to disclose.

## Funding

This work was supported by 10.13039/100008322CSL Behring GmbH.

## Declaration of competing interest

Jens Greve has received speaker/consultancy fees from CSL Behring, Kalvista, Takeda, Otsuka and BioCryst. He has also received funding to attend conferences/educational events from CSL Behring and Takeda. He has participated as an investigator in a clinical trial/registry for CSL Behring, Pharvaris, BioCryst and Takeda. Nils Picker is an employee of GIPAM GmbH, which received funding from CSL Behring for this study. Thomas Wilke holds equity in GIPAM GmbH. Fabian Jülich is an employee of CSL Behring. At the time the study was conducted, Verena Donatz was employed by CSL Behring. Ulf Maywald reports consulting fees from CSL Behring, outside the submitted work.
