# Supplementary material for: Long-term prophylactic treatment and related real-world outcomes of patients with hereditary angioedema in Germany: A retrospective analysis of German claims and medical chart data
Source: World Allergy Organ J. 2026 Sep 22;19(10):101469. doi: 10.1016/j.waojou.2026.101469 (PMC13631649; doi:10.1016/j.waojou.2026.101469)
Supplement: Supplementary file 1 [file mmc1.docx]

**Supplemental Material**

Supplemental Figures

Figure S 1. MCR – Distribution of HAE attacks requiring treatment in the first year since start of LTP therapy

**Abbreviations:** HAE, hereditary angioedema; LTP, long-term prophylaxis; ODT, on-demand treatment

Figure S 2. Cumulative healthcare cost since start of LTP treatment per person-year

**Abbreviations:** LTP, long-term prophylaxis; ODT, on-demand treatment
